# Validation in the ESPOIR cohort of vitamin K-dependent protein S (PROS) as a potential biomarker capable of predicting response to the methotrexate/etanercept combination

**DOI:** 10.1186/s13075-022-02762-5

**Published:** 2022-03-21

**Authors:** Olivier Vittecoq, Clément Guillou, Julie Hardouin, Baptiste Gerard, Francis Berenbaum, Arnaud Constantin, Nathalie Rincheval, Bernard Combe, Thierry Lequerre, Pascal Cosette

**Affiliations:** 1 Rouen University Hospital, Department of Rheumatology & CIC-CRB1404, Normandie Univ, UNIROUEN, F 76000 Rouen, France; 2 Inserm 1234 (PANTHER), F76000 Rouen, France; 3 Normandie Univ, PISSARO Proteomics Facility, IRIB, 76130 Mont-Saint Aignan, France & PBS-UMR6270 CNRS, FR3038 CNRS, 76130 Mont-Saint Aignan, France; 4 Department of Rheumatology, AP-HP Saint-Antoine Hospital, Sorbonne University, Inserm CRSA, Paris, France; 5 Rheumatology Department, Toulouse University Hospital, UMR 1043 & Université Toulouse III-Paul Sabatier, Toulouse, France; 6 Unit of Statistics, Institute of Clinical Research EA2415, Montpellier University, Montpellier, France; 7 Rheumatology Department, CHU Montpellier, Montpellier University, Montpellier, France

**Keywords:** PROS, CO7, Prediction, Rheumatoid arthritis, Etanercept, Adalimumab, Response, Biomarker

## Abstract

**Background:**

To validate the ability of PROS (vitamin K-dependent protein S) and CO7 (complement component C7) to predict response to the methotrexate (MTX)/etanercept (ETA) combination in rheumatoid arthritis (RA) patients who received this therapeutic combination in a well-documented cohort.

**Method:**

From the ESPOIR cohort, RA patients having received the MTX/ETA or MTX/adalimumab (ADA) combination as a first-line biologic treatment were included. Serum concentrations of PROS and CO7 were measured by ELISA prior to the initiation of ETA or ADA, at a time where the disease was active (DAS28 ESR > 3.2). The clinical efficacy (response/non-response) of both combinations has been evaluated after at least 6 months of treatment, according to the EULAR response criteria with some modifications.

**Results:**

Thirty-two were treated by MTX/ETA; the numbers of responders and non-responders were 24 and 8, respectively. Thirty-three patients received the MTX/ADA combination; 27 and 5 patients were respectively responders and non-responders. While there were no differences for demographic, clinical, biological, and X-rays data, as well as for CO7, serum levels of PROS tended to be significantly higher in responders to the MTX/ETA combination (*p* = 0.08) while no difference was observed in the group receiving MTX/ADA. For PROS, the best concentration threshold to differentiate both groups was calculated at 40 μg/ml using ROC curve. The theranostic performances of PROS appeared better for the ETA/MTX combination. When considering the response to this combination, analysis of pooled data from ESPOIR and SATRAPE (initially used to validate PROS and CO7 as potential theranostic biomarkers) cohorts led to a higher theranostic value of PROS that became significant (*p* = 0.009).

**Conclusion:**

PROS might be one candidate of a combination of biomarkers capable of predicting the response to MTX/ETA combination in RA patients refractory to MTX.

**Trial registration:**

ClinicalTrials.gov identifiers: NCT03666091 and NCT00234234.

## Background

Rheumatoid arthritis (RA) is one of the most common chronic inflammatory rheumatism affecting 0.8% of the world’s population. To face this pathology, several molecules targeting pro-inflammatory compounds of the immune system have been developed successfully. They are authorized for the treatment of RA, and the most prescribed are biologic DMARDs (bDMARDs) targeting cytokines. However, clinicians observe that about 30–40% of treated patients do not respond to these biomolecules. Given the heterogeneity of patients’ response to the different treatments and the adverse effects related to these treatments (infections, cancers, allergic reactions), but also the increasing number of molecules available for sale, the ability to predict response to treatment has become a very important issue in RA.

So far, only a few studies backed by proteomic analysis have sought to identify predictive markers of response to treatments. Trocmé et al. identified, by a SELDI approach, 5 proteins differentially expressed between patients responding and not responding to infliximab, responders showing overexpression of apolipoprotein A1, and a subexpression of platelet factor 4 [1]. Besides, prediction of response to etanercept (ETA) was evaluated by three studies using proteomic tools. The first one based on a multiparametric approach combining antigenic and cytokine profiles in 3 different cohorts (21 to 43 patients) enabled the identification of a combination of 24 biomarkers whose performances (VPP of 58 to 72% and VPN from 63 to 78%) were limited [2]. A second study evaluated the interest of a chip of 12 cytokines in 33 proven RA patients. It showed that high serum concentrations of MCP-1 and epidermal growth factor (EGF) were associated with a good response to treatment [3]. In the third study using 2D gel electrophoresis and mass spectrometry for analysis of 50 sera from RA patients treated by ETA, four proteins (haptoglobin-alpha 1; haptoglobin-alpha 2, vitamin D-binding protein, apolipoprotein C-III) were differentially expressed in responders and non-responders [4]. More recently, a multivariate model combining three biomarkers (prealbumin, platelet factor 4, and S100A2) was proposed to accurately predict the response of RA patients to TNF inhibitors [5]. Considering both proteomic data and those obtained from complementary approaches also based on no a priori knowledge such as pharmacogenetics, transcriptomics, and metabolomics, the panel of candidate markers for predicting the response to TNF-alpha antagonists is particularly high [6]. However, except for the interferon signature, there is no robust biomarker able to predict response to TNF-antagonists [6]. Indeed, the main issue is the absence of replication of these data in independent populations. Thus, to date, there is no tool available to predict the response to a biological product prior to its administration.

In a recent study, we have identified potential predictive markers of the response to the MTX/ETA combination in RA patients. In this regard, from a cohort of 22 patients with active RA that was treated with the MTX/ETA combination, a blood sample was collected prior to treatment for biomarkers research. By comparing the expression profiles of serum proteins of responding and non-responding patients by differential proteomic analysis (label-free approach), 12 proteins have been shown to be regulated differently depending on the patient’s response status to the treatment. Independent tests on a new cohort of 20 patients confirmed these results by two orthogonal approaches (ELISA tests and label-free analyses). Among these 12 biomarkers, 2 proteins, PROS (vitamin K-dependent protein S) and CO7 (complement component C7), were particularly discriminating. With quantifications by ELISA of these 2 proteins, it was possible to determine, via ROC curves, the concentration thresholds associated with an appropriate classification of the patients as responders or non-responders. By combining the 2 concentration thresholds, the sensitivity and specificity were respectively 75% and 100% [7]. In addition, in this combination of 12 molecules, the protein S100A9 represented another interesting biomarker candidate of MTX/ETA response in RA [8]. Nevertheless, the theranostic value of serum S100A9 was not confirmed in a large UK RA cohort treated by ETA [9].

Now, the theranostic interest of this set of 2 protein biomarkers requires to be replicated in an enlarged independent RA population. So, the main objective of this work was to validate the ability of PROS and CO7 proteins to predict response to the MTX/ETA combination in patients who received this therapeutic combination in the ESPOIR cohort. The secondary objective was to determine whether the theranostic interest of this combination of 2 proteins, PROS and CO7, is specific to MTX/ETA combination by testing it in patients who received a MTX/adalimumab (ADA) in the ESPOIR cohort.

## Methods

### Patients

The present study is based on subgroups of RA patients from the ESPOIR cohort, a large French multicentric, longitudinal, and prospective cohort [10]. Briefly, 813 patients who had undifferentiated arthritis or RA, of less than 6 months disease duration, DMARD, and steroid naïve, were included and followed during at least 10 years. RA patients who met the ACR/EULAR 2010 classification criteria [11], with an active disease defined by a DAS28-ESR > 3.2, and having received, after failure of one or more conventional DMARDs (cDMARDs), treatment with MTX/ETA or MTX/ADA for at least 6 months, were integrated in this study. Among the 104 patients who were treated with ETA and 98 patients with ADA, an average of 70–80% of them received anti-TNF in combination with MTX.

### Data collected

Demographic data (age, sex, body mass index, tobacco use), as well as characteristics of these two groups of patients [age of rheumatism, number of painful joints (out of 28), number of swollen joints (out of 28), DAS28-ESR, overall assessment of the disease by the patient using visual analog scale (0–10), functional impairment (HAQ index), autoimmune profile (rheumatoid factor and anti-CCP), systemic inflammation (CRP and ESR levels), conventional treatments already received, weekly dose of MTX, daily dose of prednisone], have been collected during the visit preceding the initiation of ETA or ADA.

DAS28-ESR was also calculated during a visit to evaluate treatment response (between 6 and 12 months of treatment depending on the date of the visit, as the response can only be evaluated after at least 6 months of consecutive treatment).

### Biological methodology

#### Serum samples

The analysis has been focused on a single serum sample, i.e., the one collected during the visit preceding the introduction of anti-TNF (which may take place several weeks after the visit but less than 6 months) at a time where the disease was active (DAS28 ESR > 3.2) during serum sampling. The doses of MTX and glucocorticoids had to be stable before the visit to collect the serum sample. The dose of prednisone was less than or equal to 10 mg/day. One biological resources center (J. Benessiano, Paris-Bichat) was in charge of centralizing and managing biological data collection. S. Martin (Bichat Hospital, Paris) did all the central dosages of CRP, IgM rheumatoid factor, and anti-CCP antibodies.

Sera collected from patients included in the SATRAPE study (ClinicalTrials.gov identifier: NCT00234234), where some of them were used for validation of the theranostic combination in the previous work [7], have also been analyzed herein with those coming from the ESPOIR cohort to have a significant sample size to draw relevant conclusions.

#### ELISA

Serum concentrations of PROS and CO7 have been measured prior to the initiation of ETA or ADA treatment by ELISA using the supplier’s instructions (USCNK from USA and EIAAB from China, respectively).

#### Criterion of judgment

The clinical efficacy of the MTX/ETA and MTX/ADA combinations has been evaluated after at least 6 months of treatment. The response/non-response have been defined according to the EULAR response criteria with some modifications. Indeed, among the moderate responders, patients exhibiting a response close to good response were included in the responder group while those having a response close to non-response were integrated in the non-responder group. Thus, in this context of personalized medicine where the interest is to identify non-responders or good responders, the other moderate responders were not analyzed in the present study (see the “Statistical analysis” section).

### Statistical analysis

Among moderate responders, those having a delta DAS28 ESR > 1.2 and a final DAS 28 between 3.2 and 5.1 were considered as responders (*n* = 3 for ADA and *n* = 6 for ETA); patients with a delta DAS28 ESR between 0.6 and 1.2 and a final DAS 28 ESR between 3.2 and 5.1 were categorized as non-responders (*n* = 1 for ADA and ETA). Other subgroups (delta DAS28 ESR between 0.6 and 1.2 and final DA28 < or = 3.2; delta DAS28 ESR > 1.2 and final DAS28 > 5.1) remained qualified as moderate responders and were not included in the analysis as stated previously.

The unpaired *t*-test (with Welch correction) was used to compare the population characteristics and protein levels between the 2 groups (responder/non-responder) for each drug combination (MTX/ETA and MTX/ADA). Receiver operating characteristic (ROC) curves were performed to determine the best concentration threshold able to discriminate responders and non-responders in ETA/MTX and ADA/MTX combinations. Performances in terms of sensitivity, specificity, and positive and negative predictive values were calculated.

The same approach was carried out using pooled data from the ESPOIR cohort and those obtained from the cohort of 16 patients used to evaluate by ELISA the two highly discriminating proteins, PROS and CO7, in the previous work [7].

The ROC curves were calculated using Prism/GraphPad software, and the predictive accuracy of each protein, as well as the combination of both, was assessed using the area under the ROC curve. The calculated thresholds resulting from ROC curves analyses allowed to categorize patients into responders (concentration above calculated threshold) and non-responders.

## Results

### Populations studied

**Fig. 1:**
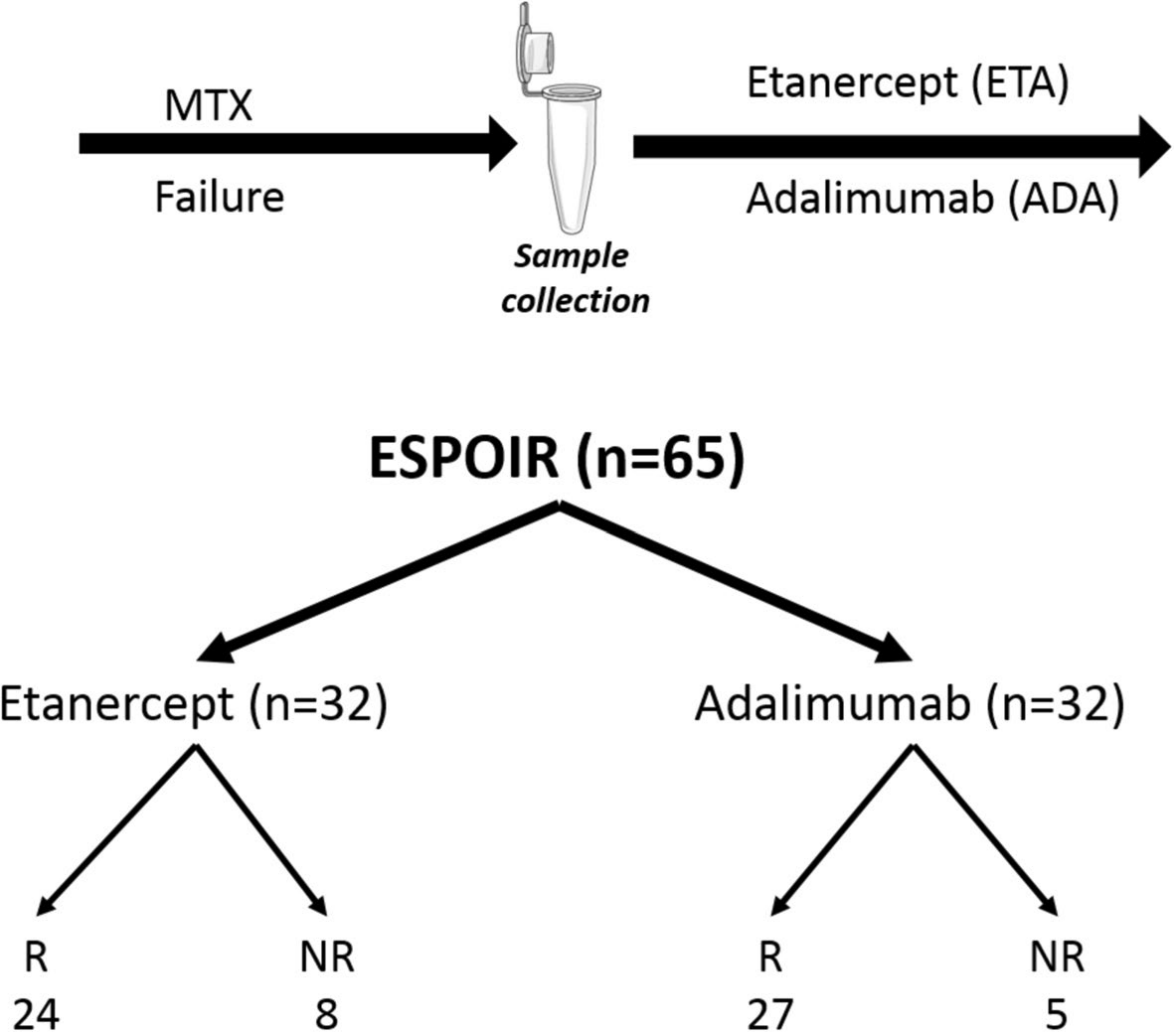
Schematic view of the study and populations of subgroups having received the different bDMARDs

From this analysis that was carried out using the ESPOIR database, only 65 fulfilled the following criteria: TNF-blocking agent in combination with MTX, biologic naïve, DAS28 ESR > 3.2 at ETA (or ADA) initiation, serum available during the 6 months preceding the introduction of anti-TNF, and assessment of the clinical response as stated in the “Biological methodology” section. Among these 65 patients, 32 were treated by MTX/ETA; the numbers of responders and non-responders were 24 and 8, respectively. Thirty-three patients received the MTX/ADA combination and 27 and 5 patients were responders and non-responders according to the EULAR criteria with some modifications as stated in the “Statistical analysis” section (Fig. 1).

For the pooled analysis, data from 9 responders and 7 non-responders of the population used for the first validation of the theranostic combination [7] were integrated.

### Characteristics of the population

Demographic, clinical, biological, radiological, and therapeutic data are summarized in Table 1. No statistical differences were observed between responder and non-responder groups for each drug combination. Characteristics of RA patients from the SATRAPE study have already been published [7].

**Table 1:** Characteristics of the whole population studied and of each subgroup treated by methotrexate/etanercept or methotrexate/adalimumab according to the responder/non-responder status in the ESPOIR cohort

		Whole population	Patients under MTX/etanercept		Patients under MTX/adalimumab	
			Responders (*n*=24)	Non responders (*n*=8)	Responders (*n*=27)	Non responders (*n*=5)
Age (years)		49 ± 2	49 ± 3	53 ± 5	48 ± 2	52 ± 6
Female/male ratio		51/13	18/6	8/0	22/5	3/2
Body mass index (kg/m^2^)		24.4 ± 0.6	24.6 ± 0.7	26.9 ± 2.4	24.3 ± 1	20.5 ± 0.7
Tobacco user (%)		20%	25%	13%	22%	0%
Disease duration (months)		32 ± 3	35 ± 4	46 ± 8	26 ± 4	21 ± 7
ACR/EULAR criteria fullfillment (%)		100%	100%	100%	100%	100%
ACR 1987 criteria fulfillment (%)		88%	92%	75%	85%	100%
Share epitope (at least one) (%)		64%	63%	63%	52%	60%
DAS 28 ESR		5.242 ± 0.141	5.279 ± 0.248	4.780 ± 0.290	5.417 ± 0,217	4.864 ± 0.573
Tender joint count/28 (*n*)		10 ± 1	10 ± 1	10 ± 3	10 ± 1	11 ± 4
Swollen joint count/28 (*n*)		7 ± 1	7 ± 1	4 ± 1	8 ± 1	5 ± 1
ESR 1st hour (mm)		28 ± 3	27 ± 4	32 ± 11	26 ± 5,0	36 ± 8
CRP (mg/L)		27.1 ± 5.0	38.9 ± 10.8	30.0 ± 17.7	18.6 ± 4.6	12.4 ± 7.2
Global activity VAS (by patient)		59.5 ± 2.7	56.3 ± 5.0	53.4 ± 5.0	64.6 ± 3.9	56.2 ± 3.9
Rheumatoid factor IgM	Positivity (%)	73%	83%	63%	67%	80%
	Titer (IU/mL)	202 ± 91	360 ± 240	72 ± 37	115 ± 33	123 ± 92
Anti-CCP	Positivity (%)	64%	71%	38%	63%	80%
	Titer (AU/mL)	1013 ± 250	1533 ± 589	207 ± 115	760 ± 228	1179 ± 809
HAQ (/3)		1.04 ± 0.08	1.05 ± 0.12	1.03 ± 0.21	1.02 ± 0.11	1.15 ± 0.34
Total Sharp score		5.7 ± 1.2	8.9 ± 2.7	2.9 ± 1.7	4.6 ± 1.2	1.2 ± 1.2
Erosion Sharp score		2.1 ± 0.7	3.5 ± 1.8	1.4 ± 1.2	1.4 ± 0.6	0.7 ± 0.7
Corticoids dose (mg/day)		4.4 ± 0.6	4.2 ± 0.9	4.1 ± 1.5	4.7 ± 0.9	3.4 ± 1.7
MTX dose (mg/week)		15.9 ± 0.6	17.2 ± 0.9	16.9 ± 1.0	14.3 ± 0.9	15.0 ± 1.8

Data are expressed in mean+/− standard deviation unless indicated otherwise

### Theranostic value of PROS and CO7 in the ESPOIR cohort

**Fig. 2:**
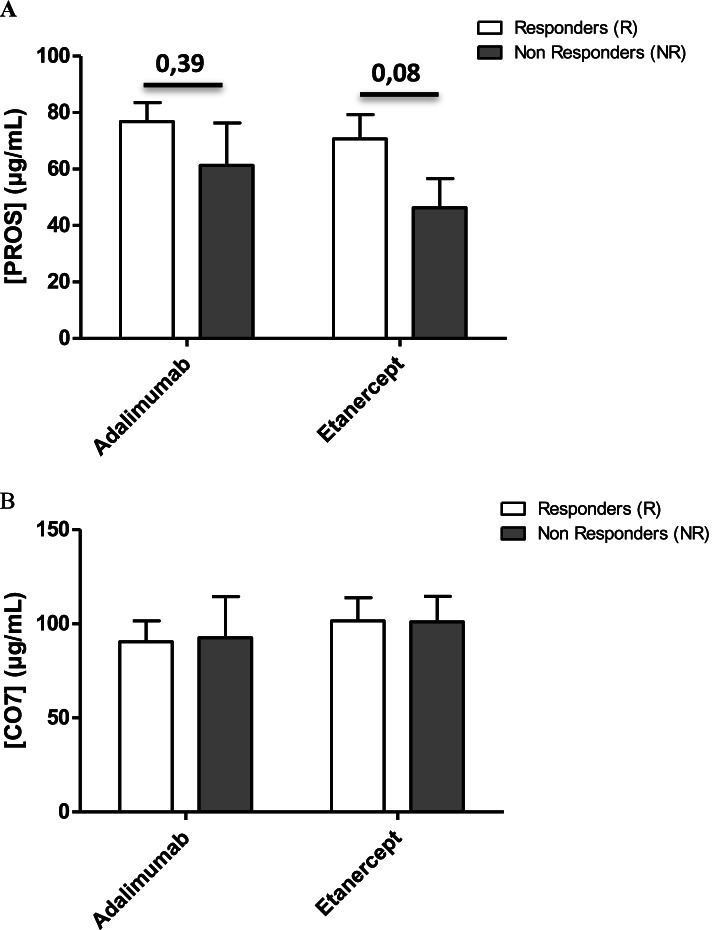
Protein levels for the different classes of patients. Serum PROS (**a**) or CO7 (**b**) concentrations in responders (*n*=24 for ETA/MTX and *n*=27 for ADA/MTX) and non-responders (*n*=8 for ETA/MTX and *n*=5 for ADA/MTX) prior to methotrexate/etanercept or methotrexate/adalimumab initiation in RA patients who have failed to methotrexate in the ESPOIR cohort

In the ESPOIR cohort, serum levels of PROS tended to be significantly higher in responders to the MTX/ETA combination (*p* = 0.08) (Fig. 2a) while no difference was observed in the group receiving MTX/ADA. Concerning the CO7 protein, whatever the combination (MTX/ETA or MTX/ADA), their serum concentrations were comparable between responders and non-responders (Fig. 2b).

**Fig. 3:**
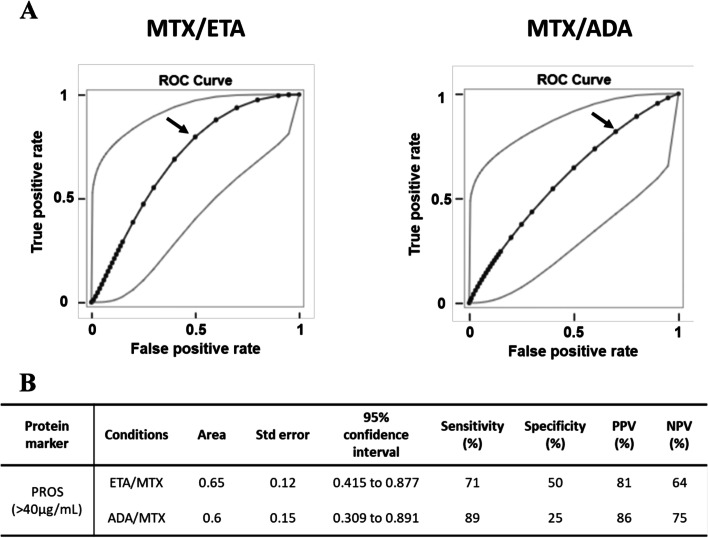
Determination of concentration threshold for PROS using receiver operating characteristic (ROC) from data of the ESPOIR cohort prior to treatment with etanercept/methotrexate or adalimumab/methotrexate. **a** ROC curve averaging of PROS prior to MTX/ETA treatment (left) or MTX/ADA combination (right). Gray line corresponds to 95% confidence interval. Black arrow corresponds to our best threshold (40 μg/mL). **b** Table showing the different parameters resulting from ROC curve analysis for each drug combination (MTX/ETA or MTX/ADA)

For PROS, the best concentration threshold has been determined using ROC curve (AUC = 0.65) to differentiate responders from non-responders in each drug combination; it was calculated at 40 μg/ml (Fig. 3a). The theranostic performances of PROS appear better for the ETA/MTX combination (Fig. 3b). Since no differences were observed between responders and non-responders in terms of clinical features at baseline for both drug combinations, no adjustment was required for this analysis.

### Theranostic value of PROS, CO7, and PROS/CO7 combination in both populations when considering the response to MTX/ETA combination

**Fig. 4:**
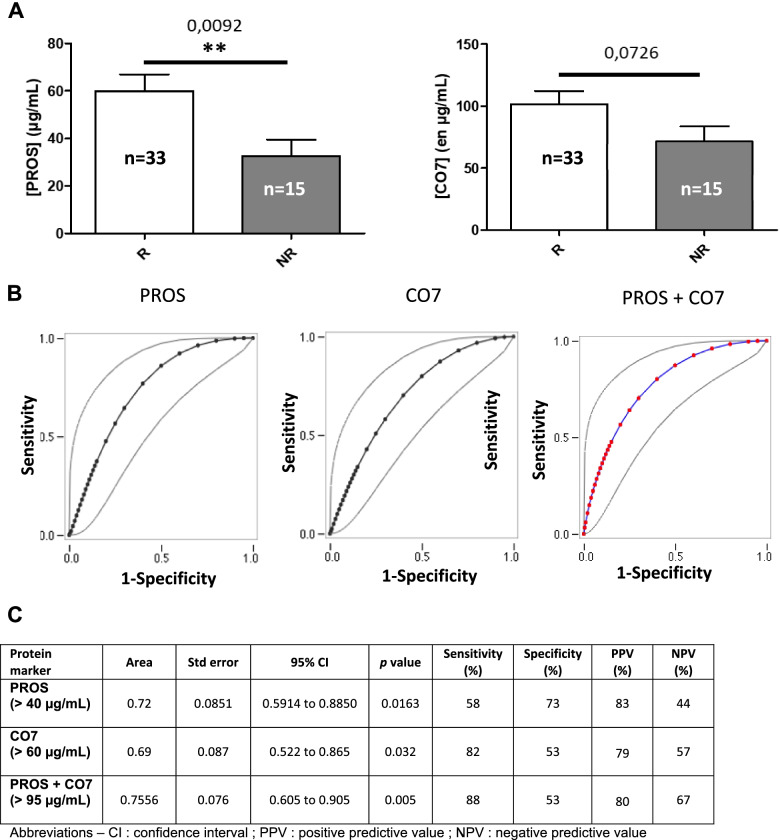
Determination of concentration threshold for PROS, CO7 and PROS + CO7 combination using receiver operating characteristic (ROC) from data of both cohorts prior to treatment with etanercept and methotrexate. **a** Serum PROS (left) or CO7 (right) concentrations in responders (*n*=33) and non-responders (*n*=15) prior to etanercept/methotrexate initiation in RA patients who have failed to methotrexate in the 2 cohorts. **b** ROC curve averaging of PROS (left), CO7 (middle) and PROS+ CO7 (right) prior to MTX/ETA treatment; gray line corresponds to 95% confidence interval. **c** Table showing the performances of PROS, CO7, and PROS + CO7 combination resulting from ROC curve analysis for prediction of response to MTX/ETA treatment

Analysis of pooled data obtained from both populations led to a higher theranostic value of PROS that became significant while only a trend was observed when applied to ESPOIR cohort (*p* = 0.009 versus 0.08) (Fig. 4a). Indeed, AUC of ROC curve is better (0.72 vs 0.65) and, using the same threshold (> 40 μg/ml), the specificity is also better (73% versus 50%). But, although the predictive value of CO7 for the response to ETA/MTX treatment remained limited, combining the concentration threshold of each protein led to a better AUC (0.75) and a right classification, in terms of positive- and negative-predictive values as illustrated in Fig. 4b, c. Once again, no adjustment was made for this analysis.

## Discussion

Because of the lack of tool capable of predicting the response/non-response to bDMARDs, practitioners currently perform an empirical choice between different treatments. Thus, accurate prediction of bDMARDs responses can provide valuable information on effective drug selection. Anti-TNF drugs are generally prescribed as a second-line treatment after methotrexate. Until now, a large panel of biomarkers has been identified as potential predictive factors of response to TNF-antagonists but almost none were really replicated in an independent population Therefore, the objective of this study was to validate the theranostic value of 2 biomarkers by testing their concentrations in serum samples from patients receiving the MTX/ETA or MTX/ADA combination in the ESPOIR cohort, in a well-defined condition which is failure to conventional DMARDs.

While CO7 seems to have no predictive value of response to ETA/MTX in this population, PROS has a certain relevance as a theranostic marker, even though the statistical significance of this finding is not achieved. Indeed, the potential theranostic interest of PROS has been highlighted since higher serum levels have been detected in biologic naïve patients who were responders to MTX/ETA, that is in concordance with our previous results [7]. Despite a lack of statistical power, probably due to the low number of patients in the non-responders group, we can consider that the theranostic value of PROS has been, in part, replicated. To overcome this issue, we have pooled data obtained from 2 different populations including that used for the validation of the set of proteins we had identified in the previous work [7]. With such an approach that allowed to make the sample size of the non-responder group more appropriate, the theranostic value of PROS appears more robust. More importantly, the addition of CO7 to PROS, when both proteins are considered with their own concentration threshold related to the better classification, improves the theranostic performances of PROS.

Taken together, these findings might suggest that high levels of PROS would lead to the initiation of ETA (rather than adalimumab since there was no difference between responders and non-responders with this molecule) after failure of MTX. In other words, faced to a RA patient refractory to cDMARDs, when we consider 2 TNF-antagonists (ETA and ADA), the theranostic value of PROS is more specific to ETA. That of the PROS/CO7 combination might be more relevant. Such data cannot be extrapolated to other profiles of patients, particularly those who had an inadequate response to one or several bDMARDs.

To our knowledge, to date, there is no biomarker for use in routine clinical practice for predicting at an individual patient level the response in a drug-specific of even class-specific manner. Indeed, a recent meta-analysis focused on assessment of prediction of treatment response in early RA was limited by the availability of only a small number of external validation studies in this topic. It suggested that there was, in general, insufficient evidence that the effect of treatment depended on baseline characteristics [12], even though some preliminary studies suggest that, in RA patients having an inadequate response to MTX, those having a disease of limited duration associated with high levels of anti-CCP antibodies and/or with presence of shared epitope, had higher efficacy responses versus ADA after 24 weeks [13, 14].

## Conclusions

PROS might be one candidate of a combination of biomarkers capable of predicting the response to MTX/ETA combination in RA patients refractory to MTX. However, when considered alone, its theranostic value, which has been replicated in an independent cohort, remains limited. Other biomarkers of this combination might be identified by integrative biology which is a fast-expanding field. In this respect, some machine learning approaches, either based on clinical profiles with additional genetic information or on multiomics signatures (transcription and/or DNA methylome in peripheral blood mononuclear cells, monocytes, and CD4+ T cells), have shown encouraging findings in guiding treatment decisions and particularly to identify non-responders to TNF-blocking or to accurately predict response before ADA and ETA treatment, respectively [15, 16]. We can hypothesize that the performances of those models might be improved by additional data of biomarkers such as PROS and to a lesser degree CO7.

## Data Availability

The datasets used and/or analyzed during the current study are available from the corresponding author on reasonable request. All data and material concerning ESPOIR cohort are available in the website: http://www.lacohorteespoir.fr/.

## Abbreviations

PROS: Vitamin K-dependent protein S
CO7: Complement component C7
MTX: Methotrexate
ETA: Etanercept
ADA: Adalimumab
RA: Rheumatoid arthritis
bDMARDs: Biologic disease-modifying anti-rheumatic drugs
cDMARDs: Conventional DMARDs
DAS: Disease activity score
ESR: Erythrocyte sedimentation rate
